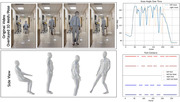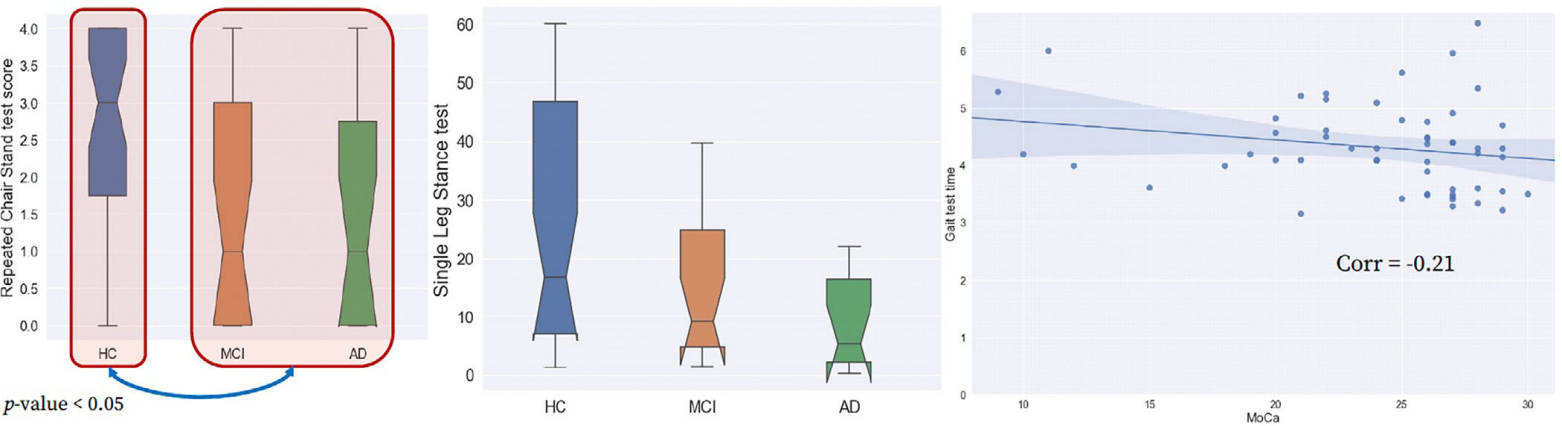# Automated Physical Performance Battery as a Digital Marker for Alzheimer’s Disease and Mild Cognitive Impairment

**DOI:** 10.1002/alz.086276

**Published:** 2025-01-03

**Authors:** Ehsan Adeli

**Affiliations:** ^1^ Stanford University, Stanford, CA USA

## Abstract

Historically, screening for incidence of AD‐related MCI or conversion from MCI to AD dementia has relied on cognitive, activities of daily living, and brain imaging measures. Limitations of this diagnostic approach include dependency on education and language, time‐consuming and costly measures, and long‐term monitoring. Emerging studies suggest that non‐tremor motor dysfunction in dementias is known to be highly associated with AD biomarkers, with signs of cognitive decline visible in gait and hand movement at various stages of the illness. With the evidence that gait and physical disturbances are early predictors of cognitive impairment and that their trajectories could readily be tracked, we utilize recent advances in computer vision (CV) to quantify mobility in a data‐driven fashion from the video‐recorded 5‐minute Short Performance Physical Battery (SPPB) tests. We use the data collected at Stanford AD Research Center and show that our CV methods can automatically reduce videos to body markers (human skeleton tracked through time) and extract several features (such as gait speed, mean torso inclination angle, double support time, gait‐summary score, etc.) and finally turn those into clinical SPPB test scores. Our initial data observed a significant difference between healthy controls (HC) and the two MCI and AD groups for the repeated chair stand test score. Similarly, an inverse correlation between the MoCa cognitive test score and the gait speed is observed. At the end of the talk, I will also discuss how this CV method for mobility can be used for detecting behavioral changes in animal AD models and implications for future human AD research.